# Quantitative measures of health policy implementation determinants and outcomes: a systematic review

**DOI:** 10.1186/s13012-020-01007-w

**Published:** 2020-06-19

**Authors:** Peg Allen, Meagan Pilar, Callie Walsh-Bailey, Cole Hooley, Stephanie Mazzucca, Cara C. Lewis, Kayne D. Mettert, Caitlin N. Dorsey, Jonathan Purtle, Maura M. Kepper, Ana A. Baumann, Ross C. Brownson

**Affiliations:** 1 Prevention Research Center, Brown School, Washington University in St. Louis, One Brookings Drive, Campus Box 1196, St. Louis, MO 63130 USA; 2 School of Social Work, Brigham Young University, 2190 FJSB, Provo, UT 84602 USA; 3 Kaiser Permanente Washington Health Research Institute, 1730 Minor Ave, Seattle, WA 98101 USA; 4 Department of Health Management & Policy, Drexel University Dornsife School of Public Health, Nesbitt Hall, 3215 Market St, Philadelphia, PA 19104 USA; 5 Brown School, Washington University in St. Louis, One Brookings Drive, Campus Box 1196, St. Louis, MO 63130 USA; 6 Department of Surgery (Division of Public Health Sciences) and Alvin J. Siteman Cancer Center, Washington University School of Medicine, 4921 Parkview Place, Saint Louis, MO 63110 USA

**Keywords:** Systematic review, Implementation science, Health policy, Policy implementation, Implementation, Public policy, Measures, Psychometric, Pragmatic

## Abstract

**Background:**

Public policy has tremendous impacts on population health. While policy development has been extensively studied, policy implementation research is newer and relies largely on qualitative methods. Quantitative measures are needed to disentangle differential impacts of policy implementation determinants (i.e., barriers and facilitators) and outcomes to ensure intended benefits are realized. Implementation outcomes include acceptability, adoption, appropriateness, compliance/fidelity, feasibility, penetration, sustainability, and costs. This systematic review identified quantitative measures that are used to assess health policy implementation determinants and outcomes and evaluated the quality of these measures.

**Methods:**

Three frameworks guided the review: Implementation Outcomes Framework (Proctor et al.), Consolidated Framework for Implementation Research (Damschroder et al.), and Policy Implementation Determinants Framework (Bullock et al.). Six databases were searched: Medline, CINAHL Plus, PsycInfo, PAIS, ERIC, and Worldwide Political. Searches were limited to English language, peer-reviewed journal articles published January 1995 to April 2019. Search terms addressed four levels: health, public policy, implementation, and measurement. Empirical studies of public policies addressing physical or behavioral health with quantitative self-report or archival measures of policy implementation with at least two items assessing implementation outcomes or determinants were included. Consensus scoring of the Psychometric and Pragmatic Evidence Rating Scale assessed the quality of measures.

**Results:**

Database searches yielded 8417 non-duplicate studies, with 870 (10.3%) undergoing full-text screening, yielding 66 studies. From the included studies, 70 unique measures were identified to quantitatively assess implementation outcomes and/or determinants. Acceptability, feasibility, appropriateness, and compliance were the most commonly measured implementation outcomes. Common determinants in the identified measures were organizational culture, implementation climate, and readiness for implementation, each aspects of the internal setting. Pragmatic quality ranged from adequate to good, with most measures freely available, brief, and at high school reading level. Few psychometric properties were reported.

**Conclusions:**

Well-tested quantitative measures of implementation internal settings were under-utilized in policy studies. Further development and testing of external context measures are warranted. This review is intended to stimulate measure development and high-quality assessment of health policy implementation outcomes and determinants to help practitioners and researchers spread evidence-informed policies to improve population health.

**Registration:**

Not registered

Contributions to the literature

- This systematic review identified 70 quantitative measures of implementation outcomes or determinants in health policy studies.
- Readiness to implement and organizational climate and culture were commonly assessed determinants, but fewer studies assessed policy actor relationships or implementation outcomes of acceptability, fidelity/compliance, appropriateness, feasibility, or implementation costs.
- Study team members rated most identified measures’ pragmatic properties as good, meaning they are straightforward to use, but few studies documented pilot or psychometric testing of measures.
- Further development and dissemination of valid and reliable measures of policy implementation outcomes and determinants can facilitate identification, use, and spread of effective policy implementation strategies.

## Background

Despite major impacts of policy on population health [1–7], there have been relatively few policy studies in dissemination and implementation (D&I) science to inform implementation strategies and evaluate implementation efforts [8]. While health outcomes of policies are commonly studied, fewer policy studies assess implementation processes and outcomes. Of 146 D&I studies funded by the National Institutes of Health (NIH) through D&I funding announcements from 2007 to 2014, 12 (8.2%) were policy studies that assessed policy content, policy development processes, or health outcomes of policies, representing 10.5% of NIH D&I funding [8]. Eight of the 12 studies (66.7%) assessed health outcomes, while only five (41.6%) assessed implementation [8].

Our ability to explore the differential impact of policy implementation determinants and outcomes and disentangle these from health benefits and other societal outcomes requires high quality quantitative measures [9]. While systematic reviews of measures of implementation of evidence-based interventions (in clinical and community settings) have been conducted in recent years [10–13], to our knowledge, no reviews have explored the quality of quantitative measures of determinants and outcomes of policy implementation.

Policy implementation research in political science and the social sciences has been active since at least the 1970s and has much to contribute to the newer field of D&I research [1, 14]. Historically, theoretical frameworks and policy research largely emphasized policy development or analysis of the content of policy documents themselves [15]. For example, Kingdon’s Multiple Streams Framework and its expansions have been widely used in political science and the social sciences more broadly to describe how factors related to sociopolitical climate, attributes of a proposed policy, and policy actors (e.g., organizations, sectors, individuals) contribute to policy change [16–18]. Policy frameworks can also inform implementation planning and evaluation in D&I research. Although authors have named policy stages since the 1950s [19, 20], Sabatier and Mazmanian’s Policy Implementation Process Framework was one of the first such frameworks that gained widespread use in policy implementation research [21] and later in health promotion [22]. Yet, available implementation frameworks are not often used to guide implementation strategies or inform why a policy worked in one setting but not another [23]. Without explicit focus on implementation, the intended benefits of health policies may go unrealized, and the ability may be lost to move the field forward to understand policy implementation (i.e., our collective knowledge building is dampened) [24].

Differences in perspectives and terminology between D&I and policy research in political science are noteworthy to interpret the present review. For example, Proctor et al. use the term implementation outcomes for what policy researchers call policy outputs [14, 20, 25]. To non-D&I policy researchers, policy implementation outcomes refer to the health outcomes in the target population [20]. D&I science uses the term fidelity [26]; policy researchers write about compliance [20]. While D&I science uses the terms outer setting, outer context, or external context to point to influences outside the implementing organization [26–28], non-D&I policy research refers to policy fields [24] which are networks of agencies that carry out policies and programs.

Identification of valid and reliable quantitative measures of health policy implementation processes is needed. These measures are needed to advance from classifying constructs to understanding causality in policy implementation research [29]. Given limited resources, policy implementers also need to know which aspects of implementation are key to improve policy acceptance, compliance, and sustainability to reap the intended health benefits [30]. Both pragmatic and psychometrically sound measures are needed to accomplish these objectives [10, 11, 31, 32], so the field can explore the influence of nuanced determinants and generate reliable and valid findings.

To fill this void in the literature, this systematic review of health policy implementation measures aimed to (1) identify quantitative measures used to assess health policy implementation outcomes (IOF outcomes commonly called policy outputs in policy research) and inner and outer setting determinants, (2) describe and assess pragmatic quality of policy implementation measures, (3) describe and assess the quality of psychometric properties of identified instruments, and (4) elucidate health policy implementation measurement gaps.

## Methods

The study team used systematic review procedures developed by Lewis and colleagues for reviews of D&I research measures and received detailed guidance from the Lewis team coauthors for each step [10, 11]. We followed the PRISMA reporting guidelines as shown in the checklist (Supplemental Table 1). We have also provided a publicly available website of measures identified in this review (https://www.health-policy-measures.org/).

For the purposes of this review, *policy* and *policy implementation* are defined as follows. We deemed public policy to include legislation at the federal, state/province/regional unit, or local levels; and governmental regulations, whether mandated by national, state/province, or local level governmental agencies or boards of elected officials (e.g., state boards of education in the USA) [4, 20]. Here, public policy implementation is defined as the carrying out of a governmental mandate by public or private organizations and groups of organizations [20].

Two widely used frameworks from the D&I field guide the present review, and a third recently developed framework that bridges policy and D&I research. In the Implementation Outcomes Framework (IOF), Proctor and colleagues identify and define eight implementation outcomes that are differentiated from health outcomes: acceptability, adoption, appropriateness, cost, feasibility, fidelity, penetration, and sustainability [25]. In the Consolidated Framework for Implementation Research (CFIR), Damschroder and colleagues articulate determinants of implementation including the domains of intervention characteristics, outer setting, inner setting of an organization, characteristics of individuals within organizations, and process [33]. Finally, Bullock developed the Policy Implementation Determinants Framework to present a balanced framework that emphasizes both internal setting constructs and external setting constructs including policy actor relationships and networks, political will for implementation, and visibility of policy actors [34]. The constructs identified in these frameworks were used to guide our list of implementation determinants and outcomes.

### Searches

Through EBSCO, we searched MEDLINE, PsycInfo, and CINAHL Plus. Through ProQuest, we searched PAIS, Worldwide Political, and ERIC. Due to limited time and staff in the 12-month study, we did not search the grey literature. We used multiple search terms in each of four required levels: health, public policy, implementation, and measurement (Table 1). Table 1 shows search terms for each string. Supplemental Tables 2 and 3 show the final search syntax applied in EBSCO and ProQuest.

**Table 1:** Search terms and strings

String	Search terms
Health	“health” OR “healthcare” OR “healthy” OR “healthier” OR “wellness”
Public policy	“policy” OR “policies” OR “law” OR “laws” OR “legislation” OR “legislative” OR “statute” OR “statutes” OR “regulation” OR “regulations” OR “regulatory” OR “executive order” OR “executive orders” OR “congress” OR “congresses” OR “congressional” OR “city council” OR “city councils” OR “county council” OR “county councils” OR mandat* OR “ordinance” OR “ordinances” OR “rule” OR “rules”
Implementation	“implement*” OR disseminat* OR “institutionalization” OR “institutionalisation” OR “integrate” OR “integrates” OR “integrated” OR “integrating” OR “integration” OR “integrations” OR “knowledge transfer” OR “knowledge exchange” OR “knowledge translation” OR “knowledge diffusion” OR “knowledge utilization” OR “research utilization” OR “innovation”
Measurement	“measure” OR “measures” OR “measurement” OR “measurements” OR “instrument” OR “instruments” OR “survey” OR “surveys” OR “questionnaire” OR “questionnaires” OR “scale” OR “scales” OR “self-report” OR “self-reports” OR “self-reported” OR “archived data” OR “archival data” OR “quantitative” OR “quantitatively” OR “inventory” OR “inventories” OR “rating” OR “ratings” OR “assessment form” OR “assessment forms” OR “evaluation form” OR “evaluation forms” OR “tool” OR “tools” OR “index” OR “indexes” OR “indices”

The authors developed the search strings and terms based on policy implementation framework reviews [34, 35], additional policy implementation frameworks [21, 22], labels and definitions of the eight implementation outcomes identified by Proctor et al. [25], CFIR construct labels and definitions [9, 33], and additional D&I research and search term sources [28, 36–38] (Table 1). The full study team provided three rounds of feedback on draft terms, and a library scientist provided additional synonyms and search terms. For each test search, we calculated the percentage of 18 benchmark articles the search captured. We determined a priori 80% as an acceptable level of precision.

### Inclusion and exclusion criteria

This review addressed only measures of implementation by organizations mandated to act by governmental units or legislation. Measures of behavior changes by individuals in target populations as a result of legislation or governmental regulations and health status changes were outside the realm of this review.

There were several inclusion criteria: (1) empirical studies of the implementation of public policies already passed or approved that addressed physical or behavioral health, (2) quantitative self-report or archival measurement methods utilized, (3) published in peer-reviewed journals from January 1995 through April 2019, (4) published in the English language, (5) public policy implementation studies from any continent or international governing body, and (6) at least two transferable quantitative self-report or archival items that assessed implementation determinants [33, 34] and/or IOF implementation outcomes [25]. This study sought to identify transferable measures that could be used to assess multiple policies and contexts. Here, a transferable item is defined as one that needed no wording changes or only a change in the referent (e.g., policy title or topic such as tobacco or malaria) to make the item applicable to other policies or settings [11]. The year 1995 was chosen as a starting year because that is about when web-based quantitative surveying began [39]. Table 2 provides definitions of the IOF implementation outcomes and the selected determinants of implementation. Broader constructs, such as readiness for implementation, contained multiple categories.

**Table 2:** Health policy implementation outcomes and determinants assessed in included measures (*N* = 70 unique measures in 66 health policy implementation studies)

Domain	Construct	Included measures (*N* = 70) *n* (%)	Definition	Source
Implementation outcomes	Acceptability	17 (24%)	Perceptions by staff in organizations mandated to implement the policy, or perceptions of other stakeholders, that the policy mandate is agreeable, palatable, or satisfactory	Proctor et al. 2011 [25]
	Adoption*	8 (11%)	Intention and initial actions of mandated organizations to revise their organizational policies to address policy mandates (not policy development or passage of bills into law).	Proctor et al. 2011 [25]
	Appropriateness	12 (17%)	“Perceived fit, relevance, or compatibility of the [policy] for a given practice setting, provider, or consumer; and/or perceived fit of the [policy] to address a particular issue or problem”; context fit	Proctor et al. 2011, pg. 69 [25]
	Costs	10 (14%)	“Cost impact of an implementation effort”	Proctor et al. 2011, pg. 69 [25]
	Feasibility	12 (17%)	“Extent to which a new [policy] can be successfully used or carried out within a given agency or setting” Level of administration required to implement a policy, often called policy automaticity	Proctor et al. 2011, pg. 69 [25] Howlett et al. 2015 [19]
	Fidelity/compliance	18 (26%)	“Degree to which a [policy] was implemented as it was prescribed” [mandated]	Proctor et al. 2011, pg. 69 [25]
	Penetration	8 (11%)	“Integration of a [policy] within a service setting and its subsystems”	Proctor et al. 2011, pg. 70 [25]
	Sustainability	1 (1%)	“Extent [new policy] is maintained or institutionalized within a service setting’s ongoing, stable operations”	Proctor et al. 2011, pg. 70 [25]
Determinants of implementation assessed	Adaptability	7 (10%)	“Degree to which an intervention can be adapted, tailored, refined, or reinvented to meet local needs”	Damschroder et al. 2009, pg. 6 [33]
	Complexity	4 (6%)	“Perceived difficulty of implementation, reflected by duration, scope, radicalness, disruptiveness, centrality, and intricacy and number of steps required to implement”	Damschroder 2009, pg. 6 [33]
	Presence of champions	3 (4%)	Field or practice leaders, people who can facilitate, and support practice change among professionals	Bullock 2019 [34], Damschroder et al. 2009 [33]
	Organizational culture and climate (general)	27 (39%)	Culture: “Norms, values, and basic assumptions of a given organization”; or Climate: “Absorptive capacity for change”, extent policy compliance will be “rewarded, supported, and expected within their organization”	Damschroder et al. 2009, pg. 8 [33] Damschroder et al. 2009, pg.8 [33]
	Policy implementation climate	16 (23%)		
	a. Goals and feedback	6 (9%)	“Degree [the policy-mandate] goals are clearly communicated, acted upon, and fed back to staff and alignment of that feedback with goals”	Damschroder et al. 2009, pg. 9 [33]
	b. Relative priority	8 (11%)	“Individuals’ shared perception of importance of the [policy] implementation within the organization”, competing priorities	Damschroder et al. 2009, pg. 8 [33]
	Readiness for implementation	43 (61%)		Damschroder et al. 2009 [33]
	a. Communication of policy	22 (31%)	Actions taken to disseminate policy requirements and guidelines to implementers.	Identified in screening [33]
	b. Policy awareness and knowledge	18 (26%)	Implementing staff/provider awareness the policy mandate exists, or knowledge of policy content	Identified in screening [33]
	c. Leadership for implementation	13 (19%)	“Commitment, involvement, and accountability of leaders and managers with the implementation”	Damschroder et al. 2009, pg. 9 [33]
	d. Training	14 (20%)	Training of staff/providers on how to implement the policy-mandated practices	Identified in screening [33]
	e. Non-training resources	19 (27%)	“Level of resources dedicated for implementation and on-going operations including money…physical space, and time” other than training resources	Damschroder et al. 2009, pg. 9 [33]
	Structure of organization	2 (3%)	“The social architecture, age, maturity, and size of an organization”	Damschroder et al. 2009, pg. 7 [33]
	Actor relationships and networks	12 (17%)	Presence and characteristics of relationships between parallel organizations that must collaborate for policy implementation to be effective	Bullock 2019 [34]
	Visibility of policy role/policy actors	7 (10%)	Perceived presence and importance of different actors pertinent to implementation of the policy	Bullock 2019 [34]
	Political will for policy implementation	8 (11%)	Societal desire and commitment to generate resources to carry out policies	Bullock 2019 [34]
	Target population characteristics	3 (4%)	Demographics, norms, neighborhood environments of population groups that affect implementation	Bullock 2019 [34]

Exclusion criteria in the searches included (1) non-empiric health policy journal articles (e.g., conceptual articles, editorials); (2) narrative and systematic reviews; (3) studies with only qualitative assessment of health policy implementation; (4) empiric studies reported in theses and books; (5) health policy studies that only assessed health outcomes (i.e., target population changes in health behavior or status); (6) bill analyses, stakeholder perceptions assessed to inform policy development, and policy content analyses without implementation assessment; (7) studies of changes made in a private business not encouraged by public policy; and (8) countries with authoritarian regimes. We electronically programmed the searches to exclude policy implementation studies from countries that are not democratically governed due to vast differences in policy environments and implementation factors.

### Screening procedures

Citations were downloaded into EndNote version 7.8 and de-duplicated electronically. We conducted dual independent screening of titles and abstracts after two group pilot screening sessions in which we clarified inclusion and exclusion criteria and screening procedures. Abstract screeners used Covidence systematic review software [40] to code inclusion as yes or no. Articles were included in full-text review if one screener coded it as meeting the inclusion criteria. Full-text screening via dual independent screening was coded in Covidence [40], with weekly meetings to reach consensus on inclusion/exclusion discrepancies. Screeners also coded one of the pre-identified reasons for exclusion.

### Data extraction strategy

Extraction elements included information about (1) measure meta-data (e.g., measure name, total number of items, number of transferable items) and studies (e.g., policy topic, country, setting), (2) development and testing of the measure, (3) implementation outcomes and determinants assessed (Table 2), (4) pragmatic characteristics, and (5) psychometric properties. Where needed, authors were emailed to obtain the full measure and measure development information. Two coauthors (MP, CWB) reached consensus on extraction elements. For each included measure, a primary extractor conducted initial entries and coding. Due to time and staff limitations in the 12-month study, we did not search for each empirical use of the measure. A secondary extractor checked the entries, noting any discrepancies for discussion in consensus meetings. Multiple measures in a study were extracted separately.

### Quality assessment of measures

To assess the quality of measures, we applied the Psychometric and Pragmatic Evidence Rating Scales (PAPERS) developed by Lewis et al. [10, 11, 41, 42]. PAPERS includes assessment of five pragmatic instrument characteristics that affect the level of ease or difficulty to use the instrument: brevity (number of items), simplicity of language (readability level), cost (whether it is freely available), training burden (extent of data collection training needed), and analysis burden (ease or difficulty of interpretation of scoring and results). Lewis and colleagues developed the pragmatic domains and rating scales with stakeholder and D&I researchers input [11, 41, 42] and developed the psychometric rating scales in collaboration with D&I researchers [10, 11, 43]. The psychometric rating scale has nine properties (Table 3): internal consistency; norms; responsiveness; convergent, discriminant, and known-groups construct validity; predictive and concurrent criterion validity; and structural validity. In both the pragmatic and psychometric scales, reported evidence for each domain is scored from poor (− 1), none/not reported (0), minimal/emerging (1), adequate (2), good (3), or excellent (4). Higher values are indicative of more desirable pragmatic characteristics (e.g., fewer items, freely available, scoring instructions, and interpretations provided) and stronger evidence of psychometric properties (e.g., adequate to excellent reliability and validity) (Supplemental Tables 4 and 5).

**Table 3:** Psychometric and Pragmatic Evidence Rating Scale (PAPERS) domains and definitions

Scale	Domain	Definition
Pragmatic criteria	Brevity	Number of items; excellent < 10 items
	Language simplicity	Readability of items, ranging from accessible only to experts (poor) to readable at or below an 8th grade level (excellent)
	Cost to use instrument	Monetary amount researchers pay to use the instrument; excellent = freely available in the public domain
	Training ease	Extent of assessor burden due to required trainings versus manualized self-training; excellent = no training required by instrument developer
	Analysis ease	Extent of assessor burden due to complexity of scoring interpretation; excellent = cutoff scores with value labels and automated calculations
Psychometric properties	Norms	A measure of generalizability based on sample size and means and standard deviations of item values
	Internal consistency	Reliability
	Convergent construct validity	Observed association in data of two theoretically related constructs, assessed through effect sizes and correlations
	Discriminant construct validity	Observed differentiation (lack of association) of two theoretically distinct constructs, assessed through effect sizes and correlations
	Known-groups validity	Extent to which groups known to have different characteristics can be differentiated by the measure
	Predictive criterion validity	Extent to which a measure can predict or be associated with an outcome measured at a future time
	Concurrent criterion validity	Correlation of a measure’s observed scores with scores from a previously established measure of the construct
	Responsiveness	Extent to which a measure can detect changes over time, i.e., clinically important not just statistically significant changes over time
	Structural validity	Structure of test covariance, i.e., extent to which groups of items increase or decrease together versus a different pattern, assessed by goodness of fit of factor analyses or principal component analyses

Lewis et al. [11], Stanick et al. [42]

Each domain is scored from poor (− 1), none/not reported (0), minimal/emerging (1), adequate (2), good (3), or excellent (4). Specific rating scales for each domain are provided in Supplemental Tables 4 and 5

### Data synthesis and presentation

This section describes the synthesis of measure transferability, empiric use study settings and policy topics, and PAPERS scoring. Two coauthors (MP, CWB) consensus coded measures into three categories of item transferability based on quartile item transferability percentages: mostly transferable (≥ 75% of items deemed transferable), partially transferable (25–74% of items deemed transferable), and setting-specific (< 25% of items deemed transferable). Items were deemed transferable if no wording changes or only a change in the referent (e.g., policy title or topic) was needed to make the item applicable to the implementation of other policies or in other settings. Abstractors coded study settings into one of five categories: hospital or outpatient clinics; mental or behavioral health facilities; healthcare cost, access, or quality; schools; community; and multiple. Abstractors also coded policy topics to healthcare cost, access, or quality; mental or behavioral health; infectious or chronic diseases; and other, while retaining documentation of subtopics such as tobacco, physical activity, and nutrition. Pragmatic scores were totaled for the five properties, with possible total scores of − 5 to 20, with higher values indicating greater ease to use the instrument. Psychometric property total scores for the nine properties were also calculated, with possible scores of − 9 to 36, with higher values indicating evidence of multiple types of validity.

## Results

### Yields

**Fig. 1:**
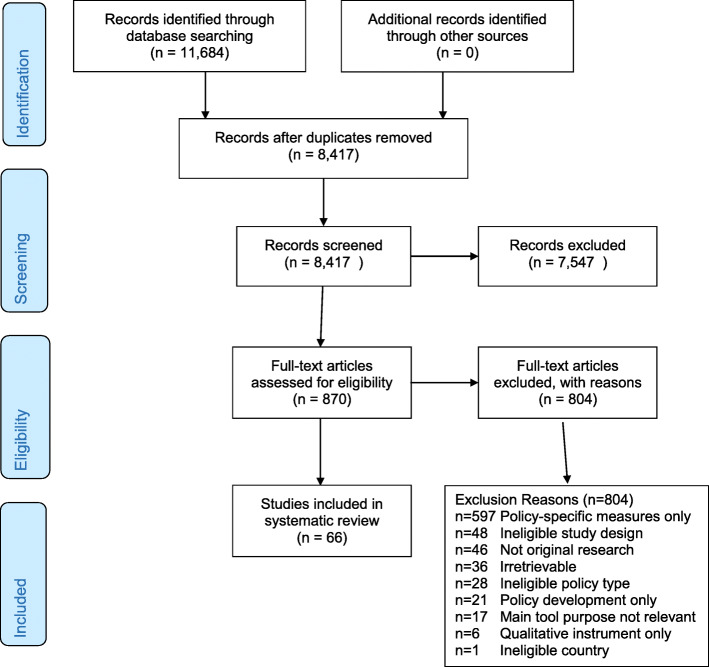
PRISMA flow diagram

The database searches yielded 11,684 articles, of which 3267 were duplicates (Fig. 1). Titles and abstracts of the 8417 articles were independently screened by two team members; 870 (10.3%) were selected for full-text screening by at least one screener. Of the 870 studies, 804 were excluded at full-text screening or during extraction attempts with the consensus of two coauthors; 66 studies were included. Two coauthors (MP, CWB) reached consensus on extraction and coding of information on 70 unique quantitative eligible measures identified in the 66 included studies plus measure development articles where obtained. Nine measures were used in more than one included study. Detailed information on identified measures is publicly available at https://www.health-policy-measures.org/.

The most common exclusion reason was lack of transferable items in quantitative measures of policy implementation (*n* = 597) (Fig. 1). While this review focused on transferable measures across any health issue or setting, researchers addressing specific health policies or settings may find the excluded studies of interest. The frequencies of the remaining exclusion reasons are listed in Fig. 1.

A variety of health policy topics and settings from over two dozen countries were found in the database searches. For example, the searches identified quantitative and mixed methods implementation studies of legislation (such as tobacco smoking bans), regulations (such as food/menu labeling requirements), governmental policies that mandated specific clinical practices (such as vaccination or access to HIV antiretroviral treatment), school-based interventions (such as government-mandated nutritional content and physical activity), and other public policies.

Among the 70 unique quantitative implementation measures, 15 measures were deemed mostly transferable (at least 75% transferable, Table 4). Twenty-three measures were categorized as partially transferable (25 to 74% of items deemed transferable, Table 5); 32 measures were setting-specific (< 25% of items deemed transferable, data not shown).

**Table 4:** Mostly transferable measures identified in studies of health policy implementation (*n* = 15)

Tool name	Number of items	Development Author, year	Empirical use Author, year Setting, country	Implementation outcomes and determinants assessed	Pragmatic PAPERS score^a^	Psychometric properties assessed
Adaptations of Evidence-Based Practices	9	Stirman et al. 2013 [44]	Lau and Brookman-Frazee 2016 [45] Mental health, USA	Fidelity/compliance, adaptability	12	Norms
Creative Climate Questionnaire	10	Ekvall 1996 [46]	Lövgren 2002 [47] Healthcare, Sweden	Organizational culture and climate	13	Norms^b^
Job Control Scale	22	Dwyer and Ganster 1991 [48]	Condon-Paoloni 2015 [49] Nutrition, Australia	Organizational culture/climate	12	Norms, internal consistency
Organizational Climate Measure	82	Patterson et al. 2005 [50]	Lau and Brookman-Frazee 2016 [45] Mental health, USA	Organizational culture/climate	10	Norms^b^
Organizational Social Context Measurement System	105	Glisson et al. 2012 [51]	Beidas et al. 2013 [52] Mental or behavioral health, USA	Organizational culture/climate, communication of policy	5	Norms, structural validity
Perceived Organizational Support Survey	8	Eisenberger et al. 1997 [53]	Eby et al. 2013 [54] Tobacco, USA	Organizational culture/climate	12	Norms, structural validity, internal consistency
Pharmaceutical Policies Survey	17	Vogler et al. 2016 [55]	Vogler et al. 2016 [55] Healthcare, Europe	Costs of implementation	11	Norms
Planning for Change Survey	4	Wanberg 2000 [56]	Eby et al. 2013 [54] Tobacco, USA	Organizational culture/climate	12	Norms, structural validity, internal consistency
Policy Coalition Evaluation Tool	15	Hardy et al. 2013 [57]	Hardy et al. 2013 [57] Community nutrition, USA	Fidelity/compliance, sustainability, readiness, organizational culture/climate, actor relationships/networks	9	Not reported
Policy Empowerment Index	12	Gavriilidis and Östergren 2012 [58]	Gavriilidis and Östergren 2012 [58] Hospitals/clinics, traditional medicine policy, South Africa	Adaptability, readiness, actor relationships, political will for implementation, target population characteristics affecting implementation	16	Not reported
Policy Implementation Barometer	10	Hongoro et al. 2018 [59]	Hongoro et al. 2018 [59] Access to care, Uganda	Appropriateness, readiness to implement	11	Norms
Policy Readiness and Stage of Change Needs Assessment	130	Roeseler et al. 2016 [60]	Roeseler et al. 2016 [60] Tobacco, USA	Adoption, fidelity/compliance	13	Norms
Rehabilitation Policy Questionnaire	7	Brämberg et al. 2015 [61]	Brämberg et al. 2015 [61] Hospitals/clinics, Sweden	Acceptability, adoption, fidelity/compliance, penetration, readiness	11	Norms
Rütten’s Health Policy Questionnaire	24	Rütten et al. 2003 [62]	Rütten et al. 2003 [62] Cancer, tobacco, physical activity, Europe (6 countries)	Acceptability, cost, org culture/climate, readiness to implement, political will implementation	15	Norms^b^
Veteran’s Administration All Employee Survey	14	Smith et al. 2017 [63]	Smith et al. 2017 [63] Mental health, USA	Organizational culture/climate	11	Norms^b^

Mostly transferable measures are defined here as those in which ≥ 75% of items can readily be used in multiple settings without change or by changing only the referent (i.e., policy name, setting)

^a^Pragmatic PAPERS score—Psychometric and Pragmatic Evidence Rating Scale [11, 41, 42], five domains assessed: brevity (score based on number of items), language simplicity, burden/ease of interpretation of scoring, and training burden, total possible score 20, higher numbers indicate greater ease to use the measure

^b^Additional subscale level psychometric properties were reported

**Table 5:** Partially transferable measures identified in studies of health policy implementation (*n* = 23)

Tool name	Number of items	Development Author, year	Empirical use Author, year Setting/topic, country	Implementation outcomes and determinants assessed	Pragmatic PAPERS score^a^	Psychometric properties assessed
Carasso User Fee Removal Questionnaire	18	Carasso et al. 2012 [64]	Carasso et al. 2012 [64] Healthcare, Zambia	Organizational culture/climate, readiness to implement	10	Norms^b^
Domain-Specific Innovativeness	6	Adapted from Goldsmith 1991 [65]	Webster et al. 2013 [66] Schools, physical activity, USA	Adoption	10	Norms, internal consistency
Evidence-Based Practice Attitude Scale	15	Aarons et al. 2010 [67]	Gill et al. 2014 [68], Beidas et al. 2013 [52] Mental health, USA, Canada	Acceptability, feasibility	12	Norms, internal consistency, structural validity^b^
Environmental Assessment Instrument	133	Lavinghouze et al. 2009 [69]	Lavinghouze et al. 2009 [69] Oral health, USA	Organizational culture/climate, champions, readiness to implement, structure of organization, actor relationships/networks, visibility of policy role/actors, political will for implementation	16	Norms
Health Enhancing Physical Activity Policy Audit Tool	75	Bull et al. 2014 [70]	Bull et al. 2015 [71] Physical activity, Europe	Readiness to implement, actor relationships/networks, political will for implementation, target population characteristics affecting implementation	12	Norms
Fall Prevention Coalition Survey	203	Schneider et al. 2016 [72]	Schneider et al. 2016 [72] Community, injury prevention, USA	Organizational culture/climate, champions, readiness to implement, actor relationships/network, visibility policy actors	7	Norms
Health Disparities Collaborative Staff Survey	21	Helfrich et al. 2007 [73]	Helfrich et al. 2007 [73] Healthcare, chronic disease, USA	Appropriateness, feasibility, adaptability, organizational climate/culture	8	Not reported
Healthy Cities Questionnaire	125	Donchin et al. 2006 [74]	Donchin et al. 2006 [74] Community, health promotion, Israel	Communication of policy, leadership for implementation, resources (non-training), actor relationships/networks, visibility of policy role/actors, political will for implementation	10	Norms^b^
Konduri Disease Registry Survey	12	Were et al. 2010 [75]	Konduri et al. 2017 [76] Hospital/clinics, tuberculosis, Ukraine	Acceptability, feasibility, readiness to implement	11	Norms, internal consistency
Local Wellness Policy Survey	39	McDonnell and Probart 2008 [77]	McDonnell and Probart 2008 [77] Schools—nutrition, physical activity, USA	Acceptability, readiness to implement, actor relationships/networks	10	Norms
Logical Assessment Matrix	9	Mersini et al. 2017 [78]	Mersini et al. 2017 [78] Nutrition, Albania	Adoption, costs of implementation, penetration, target population characteristics affecting implementation	13	Not reported
Maternal Child and Newborn Health Indicators	13	Cavagnero et al. 2008 [79]	Cavagnero et al. 2008 [79] Healthcare, global	Penetration, cost	7	Not reported
Organizational Readiness for Change	125	Lehman et al. 2002 [80]	Lau and Brookman-Frazee 2016 [45] Gill et al. 2014 [68] Mental health, USA	Organizational culture/climate	14	Norms
Perceived Attributes of Physical Activity Promotion in the Academic Classroom (PAPAC)	18	Adapted from Pankratz et al. 2002 [81]	Webster et al. 2018 [66] Schools, physical activity, USA	Appropriateness, feasibility, complexity, relative advantage	10	Norms^b^
Perceived Characteristics of Intervention Scale	20	Cook et al. 2015 [82]	Lau and Brookman-Frazee 2016 [45] Mental health, USA	Appropriateness, feasibility, adaptability, readiness to implement, relative advantage	13	Norms, structural validity^b^
Probart School Wellness Survey	39	Probart et al. 2010 [83]; Probart et al. 2008 [84]; McDonnell and Probart 2008 [77]	Probart et al. 2010 Schools, nutrition, physical activity, USA	Adoption, cost, fidelity/compliance, adaptability, organizational climate/culture	9	Norms, internal consistency
Rakic Quality and Safety Survey	50	Rakic et al. 2018 [85]	Rakic et al. 2018 [85] Healthcare QI, Bosnia and Herzegovina	Acceptability, appropriateness, feasibility, complexity, organizational culture/climate, readiness to implement, actor relationships/networks	10	Norms
Rozema Outdoor Smoking Ban Survey	14	Rozema et al. 2018 [86]	Rozema et al. 2018 [86] Schools, tobacco, Netherlands	Fidelity/compliance, organizational culture/climate, readiness to implement	14	Norms, internal consistency
School Tobacco Policy Index	40	Barbero et al. 2013 [87]	Barbero et al. 2013 [87] Schools, tobacco, USA	Fidelity/compliance, communication of policy, resources (non-training), visibility of policy role/actors	17	Norms
Specialty Care Transformation Survey	26	Williams et al. 2017 [88]	Williams et al. 2017 [88] Healthcare, access to care, USA	Appropriateness, organizational culture/climate, readiness to implement, leadership for implementation	10	Norms
Spencer Quality Improvement Survey	120	Spencer and Walshe 2009 [89]	Spencer and Walshe 2009 [89] Healthcare, quality improvement, European Union	Readiness to implement, leadership for implementation, actor relationships/networks	8	Norms
Tobacco Industry Interference Index	20	Assunta and Dorotheo 2016 [90]	Assunta and Dorotheo 2016 [90] Tobacco, Southeast Asia	Policy implementation climate, visibility of policy role/actors, political will for implementation	13	Not reported
Tummers’ Diagnosis Related Group Policy Survey 2	21	Tummers 2012 [91]	Tummers and Bekkers 2014 [92] Mental or behavioral health, Netherlands	Acceptability, adoption, appropriateness, feasibility, adaptability, champions, organizational culture/climate, relative priority, readiness to implement	11	Norms^b^

Partially transferable measures are defined here as those in which 25 to < 75% of items can readily be used in multiple settings without change or by changing only the referent (i.e., policy name, setting)

*QI* quality improvement

^a^Pragmatic PAPERS score—Psychometric and Pragmatic Evidence Rating Scale [11, 41, 42], five domains assessed: brevity (score based on number of items), language simplicity, burden/ease of interpretation of scoring, and training burden, total possible score 20, higher numbers indicate greater ease to use the measure

^b^Additional subscale level psychometric properties were reported

### Implementation outcomes

Among the 70 measures, the most commonly assessed implementation outcomes were fidelity/compliance of the policy implementation to the government mandate (26%), acceptability of the policy to implementers (24%), perceived appropriateness of the policy (17%), and feasibility of implementation (17%) (Table 2). Fidelity/compliance was sometimes assessed by asking implementers the extent to which they had modified a mandated practice [45]. Sometimes, detailed checklists were used to assess the extent of compliance with the many mandated policy components, such as school nutrition policies [83]. Acceptability was assessed by asking staff or healthcare providers in implementing agencies their level of agreement with the provided statements about the policy mandate, scored in Likert scales. Only eight (11%) of the included measures used multiple transferable items to assess adoption, and only eight (11%) assessed penetration.

Twenty-six measures of implementation costs were found during full-text screening (10 in included studies and 14 in excluded studies, data not shown). The cost time horizon varied from 12 months to 21 years, with most cost measures assessed at multiple time points. Ten of the 26 measures addressed direct implementation costs. Nine studies reported cost modeling findings. The implementation cost survey developed by Vogler et al. was extensive [53]. It asked implementing organizations to note policy impacts in medication pricing, margins, reimbursement rates, and insurance co-pays.

### Determinants of implementation

Within the 70 included measures, the most commonly assessed implementation determinants were readiness for implementation (61% assessed any readiness component) and the general organizational culture and climate (39%), followed by the specific policy implementation climate within the implementation organization/s (23%), actor relationships and networks (17%), political will for policy implementation (11%), and visibility of the policy role and policy actors (10%) (Table 2). Each component of readiness for implementation was commonly assessed: communication of the policy (31%, 22 of 70 measures), policy awareness and knowledge (26%), resources for policy implementation (non-training resources 27%, training 20%), and leadership commitment to implement the policy (19%).

Only two studies assessed organizational structure as a determinant of health policy implementation. Lavinghouze and colleagues assessed the stability of the organization, defined as whether re-organization happens often or not, within a set of 9-point Likert items on multiple implementation determinants designed for use with state-level public health practitioners, and assessed whether public health departments were stand-alone agencies or embedded within agencies addressing additional services, such as social services [69]. Schneider and colleagues assessed coalition structure as an implementation determinant, including items on the number of organizations and individuals on the coalition roster, number that regularly attend coalition meetings, and so forth [72].

### Tables of measures

Tables 4 and 5 present the 38 measures of implementation outcomes and/or determinants identified out of the 70 included measures with at least 25% of items transferable (useable in other studies without wording changes or by changing only the policy name or other referent). Table 4 shows 15 mostly transferable measures (at least 75% transferable). Table 5 shows 23 partially transferable measures (25–74% of items deemed transferable). Separate measure development articles were found for 20 of the 38 measures; the remaining measures seemed to be developed for one-time, study-specific use by the empirical study authors cited in the tables. Studies listed in Tables 4 and 5 were conducted most commonly in the USA (*n* = 19) or Europe (*n* = 11). A few measures were used elsewhere: Africa (*n* = 3), Australia (*n* = 1), Canada (*n* = 1), Middle East (*n* = 1), Southeast Asia (*n* = 1), or across multiple continents (*n* = 1).

### Quality of identified measures

**Fig. 2:**
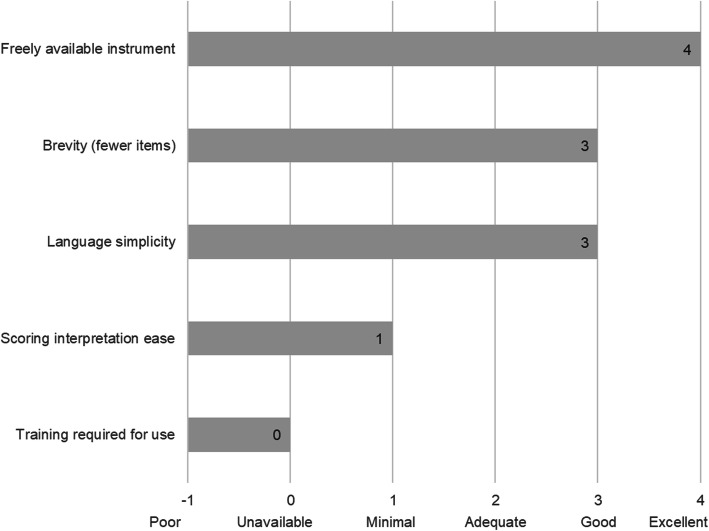
Pragmatic rating scale results across identified measures. Footnote: pragmatic criteria scores from Psychometric and Pragmatic Evidence Rating Scale (PAPERS) (Lewis et al. [11], Stanick et al. [42]). Total possible score = 20, total median score across 38 measures = 11. Scores ranged from 0 to 18. Rating scales for each domain are provided in Supplemental Table 4

Figure 2 shows the median pragmatic quality ratings across the 38 measures with at least 25% transferable items shown in Tables 4 and 5. Higher scores are desirable and indicate the measures are easier to use (Table 3). Overall, the measures were freely available in the public domain (median score = 4), brief with a median of 11–50 items (median score = 3), and had good readability, with a median reading level between 8th and 12th grade (median score = 3). However, instructions on how to score and interpret item scores were lacking, with a median score of 1, indicating the measures did not include suggestions for interpreting score ranges, clear cutoff scores, and instructions for handling missing data. In general, information on training requirements or availability of self-training manuals on how to use the measures was not reported in the included study or measure development article/s (median score = 0, not reported). Total pragmatic rating scores among the 38 measures with at least 25% of items transferable ranged from 7 to 17 (Tables 4 and 5), with a median total score of 12 out of a possible total score of 20. Median scores for each pragmatic characteristic were the same across all measures as for the 38 mostly or partially transferable measures, with a median total score of 11 across all measures.

Few psychometric properties were reported. The study team found few reports of pilot testing and measure refinement as well. Among the 38 measures with at least 25% transferable items, the psychometric properties from the PAPERS rating scale total scores ranged from − 1 to 17 (Tables 4 and 5), with a median total score of 5 out of a possible total score of 36. Higher scores indicate more types of validity and reliability were reported with high quality. The 32 measures with calculable norms had a median norms PAPERS score of 3 (good), indicating appropriate sample size and distribution. The nine measures with reported internal consistency mostly showed Cronbach’s alphas in the adequate (0.70 to 0.79) to excellent (≥ 90) range, with a median of 0.78 (PAPERS score of 2, adequate) indicating adequate internal consistency. The five measures with reported structural validity had a median PAPERS score of 2, adequate (range 1 to 3, poor to good), indicating the sample size was sufficient and the factor analysis goodness of fit was reasonable. Among the 38 measures, no reports were found for responsiveness, convergent validity, discriminant validity, known-groups construct validity, or predictive or concurrent criterion validity.

## Discussion

In this systematic review, we sought to identify quantitative measures used to assess health policy implementation outcomes and determinants, rate the pragmatic and psychometric quality of identified measures, and point to future directions to address measurement gaps. In general, the identified measures are easy to use and freely available, but we found little data on validity and reliability. We found more quantitative measures of intra-organizational determinants of policy implementation than measures of the relationships and interactions between organizations that influence policy implementation. We found a limited number of measures that had been developed for or used to assess one of the eight IOF policy implementation outcomes that can be applied to other policies or settings, which may speak more to differences in terms used by policy researchers and D&I researchers than to differences in conceptualizations of policy implementation. Authors used a variety of terms and rarely provided definitions of the constructs the items assessed. Input from experts in policy implementation is needed to better understand and define policy implementation constructs for use across multiple fields involved in policy-related research.

We found several researchers had used well-tested measures of implementation determinants from D&I research or from organizational behavior and management literature (Tables 4 and 5). For internal setting of implementing organizations, whether mandated through public policy or not, well-developed and tested measures are available. However, a number of authors crafted their own items, with or without pilot testing, and used a variety of terms to describe what the items assessed. Further dissemination of the availability of well-tested measures to policy researchers is warranted [9, 13].

What appears to be a larger gap involves the availability of well-developed and tested quantitative measures of the external context affecting policy implementation that can be used across multiple policy settings and topics [9]. Lack of attention to how a policy initiative fits with the external implementation context during policymaking and lack of policymaker commitment of adequate resources for implementation contribute to this gap [23, 93]. Recent calls and initiatives to integrate health policies during policymaking and implementation planning will bring more attention to external contexts affecting not only policy development but implementation as well [93–99]. At the present time, it is not well-known which internal and external determinants are most essential to guide and achieve sustainable policy implementation [100]. Identification and dissemination of measures that assess factors that facilitate the spread of evidence-informed policy implementation (e.g., relative advantage, flexibility) will also help move policy implementation research forward [1, 9].

Given the high potential population health impact of evidence-informed policies, much more attention to policy implementation is needed in D&I research. Few studies from non-D&I researchers reported policy implementation measure development procedures, pilot testing, scoring procedures and interpretation, training of data collectors, or data analysis procedures. Policy implementation research could benefit from the rigor of D&I quantitative research methods. And D&I researchers have much to learn about the contexts and practical aspects of policy implementation and can look to the rich depth of information in qualitative and mixed methods studies from other fields to inform quantitative measure development and testing [101–103].

### Limitations

This systematic review has several limitations. First, the four levels of the search string and multiple search terms in each level were applied only to the title, abstract, and subject headings, due to limitations of the search engines, so we likely missed pertinent studies. Second, a systematic approach with stakeholder input is needed to expand the definitions of IOF implementation outcomes for policy implementation. Third, although the authors value intra-organizational policymaking and implementation, the study team restricted the search to governmental policies due to limited time and staffing in the 12-month study. Fourth, by excluding tools with only policy-specific implementation measures, we excluded some well-developed and tested instruments in abstract and full-text screening. Since only 12 measures had 100% transferable items, researchers may need to pilot test wording modifications of other items. And finally, due to limited time and staffing, we only searched online for measures and measures development articles and may have missed separately developed pragmatic information, such as training and scoring materials not reported in a manuscript.

Despite the limitations, several recommendations for measure development follow from the findings and related literature [1, 11, 20, 35, 41, 104], including the need to (1) conduct systematic, mixed-methods procedures (concept mapping, expert panels) to refine policy implementation outcomes, (2) expand and more fully specify external context domains for policy implementation research and evaluation, (3) identify and disseminate well-developed measures for specific policy topics and settings, (4) ensure that policy implementation improves equity rather than exacerbating disparities [105], and (5) develop evidence-informed policy implementation guidelines.

## Conclusions

Easy-to-use, reliable, and valid quantitative measures of policy implementation can further our understanding of policy implementation processes, determinants, and outcomes. Due to the wide array of health policy topics and implementation settings, sound quantitative measures that can be applied across topics and settings will help speed learnings from individual studies and aid in the transfer from research to practice. Quantitative measures can inform the implementation of evidence-informed policies to further the spread and effective implementation of policies to ultimately reap greater population health benefit. This systematic review of measures is intended to stimulate measure development and high-quality assessment of health policy implementation outcomes and predictors to help practitioners and researchers spread evidence-informed policies to improve population health and reduce inequities.

## Supplementary information

**Additional file 1: Table S1**. PRISMA checklist. **Table S2**. Electronic search terms for databases searched through EBSCO. **Table S3**. Electronic search terms for searches conducted through PROQUEST. **Table S4:** PAPERS Pragmatic rating scales. **Table S5**. PAPERS Psychometric rating scales.

## Data Availability

A compendium of identified measures is available for dissemination at https://www.health-policy-measures.org/. A link will be provided on the website of the Prevention Research Center, Brown School, Washington University in St. Louis, at https://prcstl.wustl.edu/. The authors invite interested organizations to provide a link to the compendium. Citations and abstracts of excluded policy-specific measures are available on request.

## Abbreviations

CFIR: Consolidated Framework for Implementation Research
CINAHL: Cumulative Index of Nursing and Allied Health Literature
D&I: Dissemination and implementation science
EBSCO: Elton B. Stephens Company
ERIC: Education Resources Information Center
IOF: Implementation Outcomes Framework
PAPERS: Psychometric and Pragmatic Evidence Rating Scale
PRISMA: Preferred Reporting Items for Systematic Reviews and Meta-Analyses
