# Supplementary material for: Quantitative measures of health policy implementation determinants and outcomes: a systematic review
Source: Implement Sci. 2020 Jun 19;15:47. doi: 10.1186/s13012-020-01007-w (PMC7304175; doi:10.1186/s13012-020-01007-w)
Supplement: Supplementary file 1 — Additional file 1: Table S1. PRISMA checklist. Table S2. Electronic search terms for databases searched through EBSCO. Table S3. Electronic search terms for searches conducted through PROQUEST. Table S4: PAPERS Pragmatic rating scales. Table S5. PAPERS Psychometric rating scales. [file 13012_2020_1007_MOESM1_ESM.docx]

**SUPPLEMENTAL TABLES**

Supplemental Table 1. PRISMA checklist.

Supplemental Table 2. Electronic search terms for databases searched through EBSCO.

Supplemental Table 3. Electronic search terms for searches conducted through PROQUEST.

Supplemental Table 4. PAPERS Pragmatic rating scales.

Supplemental Table 5. PAPERS Psychometric rating scales.

**Supplemental Table 1. PRISMA 2009 checklist.**

| **Section/topic** | **#** | **Checklist item** | **Reported on page # in R1_Clean** |
| --- | --- | --- | --- |
| **TITLE** | | |  |
| Title | 1 | Identify the report as a literature review. | 1 |
| **ABSTRACT** | | |  |
| Structured summary | 2 | Provide a structured summary including, as applicable: background; objectives; data sources; study eligibility criteria, participants, and interventions; study appraisal and synthesis methods; results; limitations; conclusions and implications of key findings; | 2-3 |
| **INTRODUCTION** | | |  |
| Rationale | 3 | Describe the rationale for the review in the context of what is already known about your topic. | 3-5 |
| Objectives | 4 | Provide an explicit statement of questions being addressed with reference to participants, interventions, comparisons, outcomes, and study design (PICOS). | 5 |
| **METHODS** | | |  |
| Eligibility criteria | 5 | Specify study characteristics (e.g., PICOS, length of follow-up) and report characteristics (e.g., years considered, language, publication status) used as criteria for eligibility, giving rationale. | 7-8 |
| Information sources | 6 | Describe all information sources (e.g., databases with dates of coverage) in the search and date last searched. | 6-8 |
| Search | 7 | Present full electronic search strategy for at least one database, including any limits used, such that it could be repeated. | Supplemental Tables 2 & 3 |
| Study selection | 8 | State the process for selecting studies (i.e., screening, eligibility). | 7-8 |
| Risk of bias in individual studies | 9 | Describe methods used for assessing risk of bias of individual studies (including specification of whether this was done at the study or outcome level). | Not applicable |
| Risk of bias across studies | 10 | Specify any assessment of risk of bias that may affect the cumulative evidence (e.g., publication bias, selective reporting within studies). | Not assessed |
| **RESULTS** | | |  |
| Study selection | 11 | Give numbers of studies screened, assessed for eligibility, and included in the review, with reasons for exclusions at each stage, ideally with a flow diagram. | 10Figure 1 |
| Study characteristics | 12 | For each study, present characteristics for which data were extracted (e.g., study size, PICOS, follow-up period) and provide the citations. | Tables 4 & 5 |
| Synthesis of results of individual studies | 13 | For all outcomes considered (benefits or harms), present, for each study: (a) summary of results and (b) relationship to other studies under review (e.g. agreements or disagreements in methods, sampling, data collection or findings). | Not applicable |
| **DISCUSSION** | | |  |
| Summary of evidence | 14 | Summarize the main findings including the strength of evidence for each main outcome; consider their relevance to key groups (e.g., healthcare providers, users, and policy makers). | 14-16 |
| Limitations | 15 | Discuss limitations at study and outcome level (e.g., risk of bias), and at review-level (e.g., incomplete retrieval of identified research, reporting bias). | 16 |
| **CONCLUSION** | | |  |
| Conclusions | 16 | Provide a general interpretation of the results in the context of other evidence, and implications for future research. | 17 |

**Supplemental Table 2. Electronic search terms for databases searched through EBSCO.**

| String  Search Label | String  Topic | Search Terms |
| --- | --- | --- |
| S1  TI OR AB OR SU | Health | "health" OR "healthcare" OR "healthy" OR "healthier" OR "wellness" |
| S2  TI OR AB OR SU | Public Policy | "policy" OR "policies" OR "law" OR "laws" OR "legislation" OR "legislative" OR "statute" OR "statutes" OR "regulation" OR "regulations" OR "regulatory" OR "executive order" OR "executive orders" OR "congress" OR "congresses" OR "congressional" OR "city council" OR "city councils" OR "county council" OR "county councils" OR mandat* OR "ordinance" OR "ordinances" OR "rule" OR "rules" |
| S3  TI OR AB OR SU | Implementation | “implement*” OR disseminat* OR "institutionalization" OR "institutionalisation" OR "integrate" OR "integrates" OR "integrated" OR "integrating" OR "integration" OR "integrations" OR "knowledge transfer" OR "knowledge exchange" OR "knowledge translation" OR "knowledge diffusion" OR “knowledge utilization” OR "research utilization" OR "innovation" |
| S4  TI OR AB OR SU | Measurement | "measure" OR "measures" OR "measurement" OR "measurements" OR "instrument" OR "instruments" OR "survey" OR "surveys" OR "questionnaire" OR "questionnaires" OR "scale" OR "scales" OR "self-report" OR "self-reports" OR "self-reported" OR "archived data" OR “archival data” OR "quantitative" OR "quantitatively" OR "inventory" OR "inventories" OR "rating" OR "ratings" OR "assessment form" OR "assessment forms" OR "evaluation form" OR "evaluation forms" OR "tool" OR "tools" OR "index" OR "indexes" OR "indices" |
| Syntax: Row 1: (S1 AND S2 AND S3 AND S4)  Row 2: AND (S5 OR S6 OR S7 OR S8) | | |
| S5  TI OR AB | Policy near implementation | “policy” N7 (implement* OR “adoption” OR "institutionalization" OR "institutionalisation" OR "integrate" OR "integrates" OR "integrated" OR "integrating" OR "integration" OR "integrations" OR "knowledge transfer" OR "knowledge exchange" OR "knowledge translation" OR "knowledge diffusion" OR “knowledge utilization” OR "research utilization" OR "innovation") |
| S6  TI OR AB | Policies near implementation | “policies” N7 (implement* OR “adoption” OR "institutionalization" OR "institutionalisation" OR "integrate" OR "integrates" OR "integrated" OR "integrating" OR "integration" OR "integrations" OR "knowledge transfer" OR "knowledge exchange" OR "knowledge translation" OR "knowledge diffusion" OR “knowledge utilization” OR "research utilization" OR "innovation") |
| S7  TI OR AB | Mandate near implementation | “policies” N7 (implement* OR “adoption” OR "institutionalization" OR "institutionalisation" OR "integrate" OR "integrates" OR "integrated" OR "integrating" OR "integration" OR "integrations" OR "knowledge transfer" OR "knowledge exchange" OR "knowledge translation" OR "knowledge diffusion" OR “knowledge utilization” OR "research utilization" OR "innovation") |
| S8  TI OR AB | Regulation near implementation | “regulation” N7 (implement* OR “adoption” OR "institutionalization" OR "institutionalisation" OR "integrate" OR "integrates" OR "integrated" OR "integrating" OR "integration" OR "integrations" OR "knowledge transfer" OR "knowledge exchange" OR "knowledge translation" OR "knowledge diffusion" OR “knowledge utilization” OR "research utilization" OR "innovation") |

Notes: Databases searched via EBSCO: CINAHL Plus, Medline, PsychInfo

Search terms in Title (TI), Abstract (AB) or Subject Headings (SU)

Limiters: 1995 forward, Academic articles, English language

**Supplemental Table 3. Electronic search terms for searches conducted through ProQuest.**

| String  Search Label | String  Topic | Search Terms |
| --- | --- | --- |
| S1  TI OR AB OR SU | Health | "health" OR "healthcare" OR "healthy" OR "healthier" OR "wellness" |
| S2  TI OR AB OR SU | Public Policy | "policy" OR "policies" OR "law" OR "laws" OR "legislation" OR "legislative" OR "statute" OR "statutes" OR "regulation" OR "regulations" OR "regulatory" OR "executive order" OR "executive orders" OR "congress" OR "congresses" OR "congressional" OR "city council" OR "city councils" OR "county council" OR "county councils" OR mandate[*10] OR "ordinance" OR "ordinances" OR "rule" OR "rules" |
| S3  TI OR AB OR SU | Implementation | implement[*10] OR disseminat[*10] OR "institutionalization" OR "institutionalisation" OR "integrate" OR "integrates" OR "integrated" OR "integrating" OR "integration" OR "integrations" OR "knowledge transfer" OR "knowledge exchange" OR "knowledge translation" OR "knowledge diffusion" OR “knowledge utilization” OR "research utilization" OR "innovation" |
| S4  TI OR AB OR SU | Measurement | "measure" OR "measures" OR "measurement" OR "measurements" OR "instrument" OR "instruments" OR "survey" OR "surveys" OR "questionnaire" OR "questionnaires" OR "scale" OR "scales" OR "self-report" OR "self-reports" OR "self-reported" OR "archived data" OR "quantitative" OR "quantitatively" OR "inventory" OR "inventories" OR "rating" OR "ratings" OR "assessment form" OR "assessment forms" OR "evaluation form" OR "evaluation forms" OR "tool" OR "tools" OR "index" OR "indexes" OR "indices" |
| Syntax: Row 1: (S1 AND S2 AND S3 AND S4)  Row 2: AND (S5 OR S6 OR S7 OR S8) | | |
| S5  TI OR AB | Policy near implementation | “policy” N/7 (implement[*10] OR “adoption” OR "institutionalization" OR "institutionalisation" OR "integrate" OR "integrates" OR "integrated" OR "integrating" OR "integration" OR "integrations" OR "knowledge transfer" OR "knowledge exchange" OR "knowledge translation" OR "knowledge diffusion" OR “knowledge utilization” OR "research utilization" OR "innovation") |
| S6  TI OR AB | Policies near implementation | “policies” N/7 (implement[*10] OR “adoption” OR "institutionalization" OR "institutionalisation" OR "integrate" OR "integrates" OR "integrated" OR "integrating" OR "integration" OR "integrations" OR "knowledge transfer" OR "knowledge exchange" OR "knowledge translation" OR "knowledge diffusion" OR “knowledge utilization” OR "research utilization" OR "innovation") |
| S7  TI OR AB | Mandate near implementation | mandate[*10] N/7 (implement[*10] OR “adoption” OR "institutionalization" OR "institutionalisation" OR "integrate" OR "integrates" OR "integrated" OR "integrating" OR "integration" OR "integrations" OR "knowledge transfer" OR "knowledge exchange" OR "knowledge translation" OR "knowledge diffusion" OR “knowledge utilization” OR "research utilization" OR "innovation") |
| S8  TI OR AB | Regulation near implementation | “regulation” N/7 (implement[*10] OR “adoption” OR "institutionalization" OR "institutionalisation" OR "integrate" OR "integrates" OR "integrated" OR "integrating" OR "integration" OR "integrations" OR "knowledge transfer" OR "knowledge exchange" OR "knowledge translation" OR "knowledge diffusion" OR “knowledge utilization” OR "research utilization" OR "innovation") |

Notes: Databases searched via PROQUEST: PAIS Index, Worldwide Political Science Abstract, ERIC; Search terms in Title (TI), Abstract (AB) or

Subject Headings (SU); Limiters: 1995 forward, Peer Reviewed, English language, Source Type: Scholarly Journals;

Document type: Articles; MESH: health policy OR public policy

**Supplemental Table 4. PAPERS pragmatic rating scales**.

| **Brevity (length)** | |
| --- | --- |
| **-1** | Poor (P): The measure has greater than 200 items. |
| **0** | None (N): The measure is not available for use in the public domain. |
| **1** | Minimal/Emerging (M): The measure has greater than 100 items but fewer than *or equal to 200 items.* |
| **2** | Adequate (A): The measure has greater than 50 items but fewer than *or equal to 100 items.* |
| **3** | Good (G): The measure has greater than 10 items but fewer than *or equal to 50 items.* |
| **4** | Excellent (E): The measure has fewer than or equal to *10 items.* |
| **Cost** | |
| **-1** | Poor (P): The measure is extremely costly greater than or equal to $100 per use |
| **0** | None (N): The cost of the measure is unknown |
| **1** | Minimal/Emerging (M): The measure is very costly greater than or equal to $50 but < $100 per use |
| **2** | Adequate (A): The measure is somewhat costly greater than or equal to $1 but < $50 per use |
| **3** | Good (G): The measure is not costly < $1 per use |
| **4** | Excellent (E): The measure is free and in the public domain |
| **Assessor Burden (ease of training)** | |
| **-1** | Poor (P): The measure requires an external, expert administrator, with no option to self-train or for a train-the-administrator component. |
| **0** | None (N): The training and administration information for the measure is unavailable. |
| **1** | Minimal/Emerging (M): The measure requires a train-the-trainer to administer component that is specialized or includes a significant cost (greater than or equal to $100). |
| **2** | Adequate (A): The measure requires some training, in addition to a manual, and/or supervision/consultation with experts is needed to administer the measure which includes minimal cost (i.e., small consultant fee) (greater than or equal to $50 but less than $100) |
| **3** | Good (G): The measure includes a manual in order to self-train for administration and the cost for the manual is free or minimal (less than $50 but not free). |
| **4** | Excellent (E): The measure requires no training and/or has free automated administration |
| **Assessor Burden (easy of interpretation)** | |
| **-1** | Poor (P): The measure requires an expert to score and interpret, though no entity to whom to send the measure is identified, and no information on handling missing data is provided |
| **0** | None (N): The ease of interpreting the measure cannot be assessed because the measure is not in the public domain |
| **1** | Minimal/Emerging (M): The measure does not include suggestions for interpreting score ranges, no clear cut-off scores, and no instructions for handling missing data |
| **2** | Adequate (A): The measure includes a range of scores with few suggestions for interpreting them but no clear cut-off scores and no instructions for handling missing data |
| **3** | Good (G): The measure includes a range of scores with value labels and cut-off scores, but scoring requires manual calculation and/or additional inspection of response patterns or subscales, and no instructions for handling missing data are provided |
| **4** | Excellent (E): The measure includes clear cut-off scores with value labels, instructions for handling missing data are provided, and calculation of scores is automated or scores can be sent off to an identified entity for calculation with results returned |
| **Language** | |
| **-1** | Poor (P): The measure used language that was only readable by experts in its content |
| **0** | None (N): The measure was not available in the public domain and therefore the readability cannot be assessed |
| **1** | Minimal/Emerging (M): The readability of the measure was at a graduate study level (range: 17.0 and above). |
| **2** | Adequate (A): The readability of the measure was at a college level (range: 13.0 – 16.99). |
| **3** | Good (G): The readability of the measure was between an 8th and 12th grade level (range: 8.0 – 12.99). |
| **4** | Excellent (E): The readability of the measure was at or below an 8th grade level (range: 7.9 and below) |

**Supplemental Table 5. PAPERS psychometric rating scales.**

| **Reliability - Internal Consistency** | |
| --- | --- |
| -1 | Poor (P): Cronbach’s α values of **< 0.50** |
| 0 | None (N): Internal consistency measures are not applicable for this instrument **OR** classical test theory anchors are not appropriate (results reported using item response theory) **OR** α values are not yet available for the full measure scale or any associated subscales. |
| 1 | Minimal/Emerging (M): Cronbach’s α values = **0.50-0.69** |
| 2 | Adequate (A): Cronbach’s α values of = **0.70 - 0.79** |
| 3 | Good (G): Cronbach’s α values of = **0.80 - 0.89** |
| 4 | Excellent (E): Cronbach’s α values of **≥ 0.90** |
| **Norms** | |
| -1 | Poor (P): Measures of central tendency and distribution for the total score (and subscales if relevant) based only on a very small (**n < 50**) sample are available. |
| 0 | None (N): Norms not yet available. |
| 1 | Minimal/Emerging (M): Measures of central tendency and distribution for the total score (and subscales if relevant) based only on a small (**n = 50-99**) sample are available. |
| 2 | Adequate (A): Measures of central tendency and distribution for the total score (and subscales if relevant) based only on a small (**n = 100-299**) sample are available. |
| 3 | Good (G): Measures of central tendency and distribution for the total score (and subscales if relevant) based on a medium (**n = 300-499**) sample are available. |
| 4 | Excellent (E): Measures of central tendency and distribution for the total score (and subscales if relevant) based on a large (**n ≥ 500**) sample are available. |
| **Construct Validity - Convergent** | |
| -1 | Poor: **Cohen’s *d* ≤ 0.10** |
| 0 | None (N): Convergent validity measures are not applicable for this instrument OR convergent validity was not assessed. |
| 1 | Minimal/Emerging: **0.10 < Cohen’s *d* ≤ 0.20** |
| 2 | Adequate: **0.20 < Cohen’s *d* ≤ 0.50** |
| 3 | Good: **0.50 < Cohen’s *d* ≤ 0.80** |
| 4 | Excellent: **Cohen’s *d* > 0.80** |
| **Construct Validity - Divergent** | |
| -1 | Poor: **Cohen’s *d* > 0.80** |
| 0 | None (N): Discriminant validity measures are not applicable for this instrument OR discriminant validity was not assessed. |
| 1 | Minimal/Emerging: **0.50 < Cohen’s *d* ≤ 0.80** |
| 2 | Adequate: **0.20 < Cohen’s *d* ≤ 0.50** |
| 3 | Good: **0.10 < Cohen’s *d* ≤ 0.20** |
| 4 | Excellent: **Cohen’s *d* ≤ 0.10** |
| **Construct Validity – Known-Groups** | |
| -1 | Poor (P): Known-groups validity failed to be detected. |
| 0 | None (N): Known-groups validity not yet tested. |
| 1 | Minimal/Emerging (M): Statistically significant difference between groups detected, but no hypothesis tested |
| 2 | Adequate (A): Two or more statistically significant difference between groups detected, but no hypotheses tested |
| 3 | Good (G): Statistically significant difference between groups detected AND hypothesis tested |
| 4 | Excellent (E): Two or more statistically significant differences between groups detected AND hypotheses tested |
| **Criterion Validity - Predictive** | |
| Evidence of correlation (Pearson’s r) between instrument and scores on another test (measuring a distinct construct of interest or outcome) administered at some point in the future. | |
| -1 |  |
| 0 | None (N): Predictive validity not tested. |
| 1 | Minimal/Emerging (M): Pearson’s *r* = **0.10-0.29** |
| 2 | Adequate (A): Pearson’s *r* = **0.30-0.49** |
| 3 | Good (G): Pearson’s *r* = **0.50-0.69** |
| 4 | Excellent (E): Pearson’s *r* **> 0.70** |
| **Criterion Validity - Concurrent** | |
| -1 | Poor (P): Pearson’s *r* **< 0.10** |
| 0 | None (N): Concurrent validity not tested. |
| 1 | Minimal/Emerging (M): Pearson’s *r* = **0.10-0.29** |
| 2 | Adequate (A): Pearson’s *r* = **0.30-0.49** |
| 3 | Good (G): Pearson’s *r* = **0.50-0.69** |
| 4 | Excellent (E): Pearson’s *r* **> 0.70** |
| **Dimensionality – Structural Validity** | |
| Normed Fit Index = NFI ; Incremental Fit Index = IFI ; Goodness of Fit Index = GFI ; Tucker-Lewis Index = TLI; Comparative Fit Index = CFI ; Relative Noncentrality Fit Index = RNI; Standardized RMR = SRMR ; Root Mean Square Error of Approximation = RMSEA; Weighted Root Mean Residual = WRMR | |
| -1 | Poor (P): The sample consisted of less than 5 times the number of items AND exploratory factor analysis explained **< 25%** of variance **OR** |
|  | NFI **OR** IFI **OR** GFI **OR** TLI **OR** CFI **OR** RNI  **≤ 0.88 OR** SRMR **OR** RMSEA = **X ≥ 0.10 OR** WRMR **≥ 0.92** |
| 0 | None (N): No exploratory or confirmatory factor analysis has yet been performed, nor have any Item Response Theory (IRT) tests of (uni-) dimensionality have been conducted **OR** analysis has been conducted but percent variance is unexplained and cannot be calculated **OR** only principal components analysis has been conducted. |
| 1 | Minimal/Emerging (M): The sample consisted of 5 times the number of items AND exploratory factor analysis explained **< 25%** of variance **OR** |
|  | NFI **OR** IFI **OR** GFI **OR** TLI **OR** CFI **OR** RNI = **0.88 < X ≤ 0.90 OR** SRMR **OR** RMSEA = **0.08 ≤ X < 0.10 OR** WRMR = **0.90 ≤ X < 0.92** |
| 2 | Adequate (A): The sample consisted of 5 times the number of items but is less than 100 in total AND an exploratory factor analysis explained **< 50%** of variance **OR** |
|  | NFI **OR** IFI **OR** GFI **OR** TLI **OR** CFI **OR** RNI = **0.90 < X ≤ 0.95 OR** SRMR **OR** RMSEA = **0.05 ≤ X < 0.08 OR** WRMR = **0.85 ≤ X < 0.90** |
| 3 | Good (G): The sample consisted of 5 times the number of items and is greater than or equal to 100 in total OR the sample consisted of 5-7 times the number of items but is less than 100 in total AND in either case exploratory factor analysis explained  **< 50%** of variance **OR** |
|  | NFI **OR** IFI **OR** GFI **OR** TLI **OR** CFI **OR** RNI = **0.95 < X ≤ 0.97 OR** SRMR **OR** RMSEA = **0.03 ≤ X < 0.05 OR** WRMR = **0.83 ≤ X < 0.85** |
| 4 | Excellent (E): The sample consisted of 7 times the number of items and is greater than 100 in total AND an exploratory factor analysis explained **> 50%** of variance **OR** |
|  | NFI **OR** IFI **OR** GFI **OR** TLI **OR** CFI **OR** RNI  **> 0.97 OR** SRMR **OR** RMSEA = **< 0.03 OR** WRMR **< 0.83** |
| **Responsiveness** | |
| Standardized Response Mean = SRM | |
| -1 | Poor (P): SRM **< 0.10 OR** Pearson’s *r* **< 0.10** |
| 0 | None (N): The instrument has not been administered both pre- and post- implementation to evaluate sensitivity to change. |
| 1 | Minimal/Emerging (M): SRM = **0.10-0.19 OR** Pearson’s *r* = **0.10-0.29** |
| 2 | Adequate (A): SRM = **0.20-0.49 OR** Pearson’s *r* = **0.30-0.49** |
| 3 | Good (G): SRM = **0.50-0.79 OR** Pearson’s *r* = **0.50-0.69** |
| 4 | Excellent (E): SRM **> 0.80 OR** Pearson’s *r* **> 0.70** |
